# A Human ABCG2 SNP Variant, P269S (rs34678167, c.805C>T), Increases Cellular Resistance to Mitoxantrone and SN-38 Without Detectable Differences in ABCG2 Abundance in Total and Biotinylated Protein Fractions

**DOI:** 10.3390/biom16101486

**Published:** 2026-10-13

**Authors:** Shiori Sato, Megumi Tsukamoto, Kayo Ozawa, Ritsuko Imai, Hiroshi Nakagawa

**Affiliations:** 1 Department of Bioscience and Biotechnology, Graduate School of Bioscience and Biotechnology, Chubu University, 1200 Matsumoto-cho, Kasugai 487-8501, Japan; 2 Department of Biological Chemistry, Graduate School of Bioscience and Biotechnology, Chubu University, 1200 Matsumoto-cho, Kasugai 487-8501, Japan

**Keywords:** ATP-binding cassette (ABC) transporter, ABCG2, BCRP, MXR

## Abstract

ABCG2 (BCRP/MXR) is an ATP-binding cassette transporter that plays a role in cellular resistance to various anticancer agents. While several non-synonymous single-nucleotide polymorphisms (SNPs) in the ABCG2 gene have been functionally characterized, the effects of many variants remain uncertain. This study examined the human ABCG2 SNP variant P269S (rs34678167, c.805C>T) in cellular resistance to mitoxantrone and SN-38 using a Flp-In-293 controlled expression system. Quantitative real-time PCR, whole-cell Western blotting, and cell-surface biotinylation analysis showed comparable ABCG2 mRNA and total protein levels and ABCG2 abundance in the biotinylated protein fraction between ABCG2(P269S)- and ABCG2(WT)-expressing cells. In MTT assays, ABCG2(P269S)-expressing cells exhibited significantly higher EC_50_ values than ABCG2(WT)-expressing cells, with mean values approximately 3.0- and 3.5-fold higher for mitoxantrone and SN-38, respectively. Consequently, P269S is associated with increased cellular resistance to mitoxantrone and SN-38 despite no detectable differences in total ABCG2 protein abundance or abundance in the biotinylated protein fraction. Collectively, these findings underscore the utility of the Flp-In-293 system for assessing variant-specific effects of ABCG2 on cellular drug responses within a controlled background. These results support future human pharmacogenomic research on rs34678167 to determine whether this variant contributes to inter-individual differences in responses to ABCG2 substrate drugs.

## 1. Introduction

Chemotherapy is one of the main strategies used for cancer treatment. The efficacy of chemotherapy varies between patients and cancer types, with genetic polymorphisms and mutations playing key roles. Therefore, understanding the effects of genetic polymorphisms and mutations in patients with cancer is critical for achieving successful chemotherapy outcomes.

**Figure 1:**
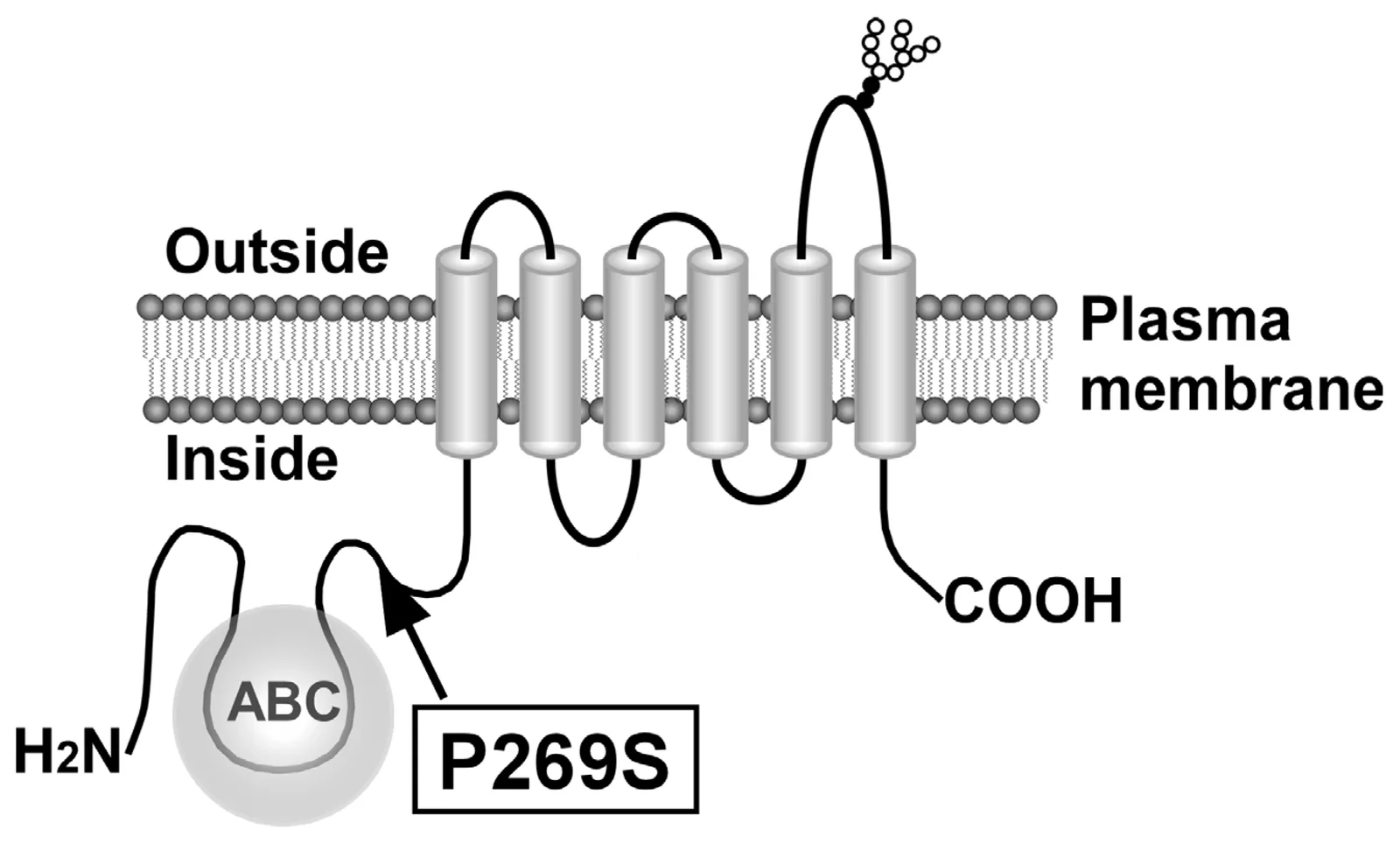
Schematic illustration of human ABCG2 and the location of SNP (rs34678167, c.805C>T, P269S). Arrow: location of SNP (rs34678167, c.805C>T, P269S); ABC, ATP-binding cassette (nucleotide-binding domain).

Some ABC transporters regulate drug concentration in the body and within cells by actively exporting substrates across biological membranes. ABCG2 (BCRP/MXR) was identified in a mitoxantrone-resistant human colorectal cancer cell line and an anthracycline-resistant breast cancer cell line [1,2,3], following the discovery of ABCB1 [4,5] and ABCC1 [6], and has also been shown to extrude anticancer drugs [7]. ABCG2, a 72 kDa glycoprotein composed of 655 amino acids, features a single ATP-binding domain at the N-terminus and a transmembrane domain at the C-terminus (Figure 1) and functions as a homodimer [8]. ABCG2 facilitates the transport of structurally diverse endobiotics, such as dehydroepiandrosterone sulfate [9], estrone-3-sulfate [9,10], lumichrome [11], porphyrins [12,13,14], and uric acid [15]. ABCG2 overexpression has been associated with increased resistance to several anticancer drugs, including camptothecin and its analogs [7,8,12,16,17], epidermal growth factor receptor tyrosine kinase inhibitors [18,19], mitoxantrone [8,20,21], and methotrexate [22,23]. In addition, recent clinical studies have indicated that ABCG2 expression levels and genetic variants can be prognostic markers for the progression and treatment response of cancers such as glioma [24], hepatoma [25], and Non-Small Cell Lung Cancer [26].

Our previous studies have demonstrated that amino acid substitutions can influence the intracellular stability and substrate specificity of ABCG2 [12,27]. In addition, our previous studies have shown that 18 non-synonymous SNPs in *ABCG2* affect the affinity of ABCG2 for porphyrins and methotrexate [12]. We have also reported that seven nonsynonymous SNPs affect the intracellular protein expression levels of ABCG2 and ABCG2-mediated drug resistance in cells [27]. Although the functional consequences of many ABCG2 SNPs remain incompletely understood, the P269S variant has previously been examined in membrane-vesicle transport systems, with comparable or reduced transport activities reported depending on the substrate and expression system [9,28,29]. In the present study, we used a controlled Flp-In-293 cellular expression system to evaluate the effect of rs34678167 (c.805C>T, P269S) on cellular resistance to mitoxantrone and SN-38, allowing direct comparison between ABCG2(WT) and ABCG2(P269S). We further examined ABCG2 mRNA levels, total protein expression, and abundance in the biotinylated protein fraction to determine whether differences in cellular resistance were associated with altered ABCG2 abundance.

## 2. Materials and Methods

### 2.1. Preparation of Plasmids Carrying the ABCG2 (P269S) Variant cDNA

To generate the expression vector pcDNA5/FRT/ABCG2 (P269S), we used site-directed mutagenesis based on the sequence information of the ABCG2 (P269S) variant obtained from the NCBI dbSNP database as previously described [12,21,27,30]. Site-directed mutagenesis was performed using PrimeSTAR^®^ Max DNA polymerase (Takara Bio Inc., Otsu, Japan) and specific primers to prepare the non-synonymous SNP variant ABCG2 (P269S) cDNA. After PCR for site-directed mutagenesis, the reaction mixture was treated with the Dpn I endonuclease to eliminate the original template plasmid pcDNA5/FRT/ABCG2 (WT) vector. The sequences of the resulting amplicons were verified using an Applied Biosystems 3130 and 3130xl Genetic Analyzer (Applied Biosystems, Foster City, CA, USA).

### 2.2. Cell Culture Conditions

Flp-In-293 cells (Invitrogen, Thermo Fisher Scientific, Waltham, MA, USA) were cultured in high-glucose Dulbecco’s Modified Eagle Medium (DMEM) supplemented with 10% heat-inactivated fetal bovine serum (FBS), 4 mM L-glutamine, 100 U/mL penicillin, 100 μg/mL streptomycin, 250 ng/mL amphotericin B, and 100 μg/mL zeocin. To select for Flp-In-293/ABCG2 (P269S) cells, 50 μg/mL hygromycin B was used instead of 100 μg/mL zeocin. The cells were maintained in a humidified incubator at 37 °C with 5% CO_2_, and their viability was assessed by a trypan blue exclusion assay using a hemocytometer. Living cells were used in all the experiments.

Zeocin was purchased from Invitrogen (Thermo Fisher Scientific, Waltham, MA, USA), while an Antibiotic-Antimycotic Mixed Stock Solution (100×) (mixture of 10,000 U/mL penicillin, 10,000 μg/mL streptomycin, and 25,000 ng/mL amphotericin B) and high-glucose DMEM were purchased from Nacalai Tesque, Inc. (Kyoto, Japan). FBS was purchased from Equitech-Bio, Inc. (Kerrville, TX, USA).

### 2.3. Generation of Cells Expressing ABCG2 (P269S) Variant

Flp-In-293 cells were plated in 35 mm dishes (TrueLine, Baton Rouge, LA, USA) at a density of 1 × 10^6^ cells per dish and precultured for 24 h. Cells were then co-transfected with pcDNA5/FRT/ABCG2 (P269S) and the Flp recombinase expression plasmid pOG44 vectors using Lipofectamine™-2000 according to the manufacturer’s protocol. After transfection, the cells were selected with 50 μg/mL hygromycin B, and the resulting hygromycin B-resistant colonies were collected, subcultured, and used as Flp-In-293/ABCG2 (P269S) cells.

Lipofectamine™-2000 and pOG44 were purchased from Invitrogen (Thermo Fisher Scientific, Waltham, MA, USA). Hygromycin B was obtained from Nacalai Tesque, Inc. (Kyoto, Japan).

### 2.4. Total RNA Preparation and First-Strand cDNA Synthesis

Flp-In-293/Mock, Flp-In-293/ABCG2(WT), and Flp-In-293/ABCG2(P269S) cells were seeded in 35 mm dishes (TrueLine, Baton Rouge, LA, USA) at a density of 1 × 10^6^ cells per dish and precultured for 3 days. Cells were collected with culture medium into 1.5 mL tubes, centrifuged (300× *g*, 5 min, 4 °C), and washed twice with 1 mL PBS(−). The resulting cell pellets were suspended in 600 μL of lysis/binding buffer from the High Pure RNA Isolation Kit (Roche Diagnostics, Mannheim, Germany) and stored at −80 °C until total RNA preparation was performed with a High-Purity RNA Isolation Kit (Roche Diagnostics). Total RNA concentration was measured using a DU640 spectrophotometer (Beckman Coulter, Fullerton, CA, USA). Total RNA was reverse transcribed using a High-Capacity cDNA Reverse-Transcription Kit (Thermo Fisher Scientific Inc., Waltham, MA, USA) according to the manufacturer’s instructions.

### 2.5. Quantitative Evaluation of ABCG2 mRNA Levels

The expression levels of ABCG2 mRNA were determined using a 7500 Fast Real-Time PCR System (Applied Biosystems), where GoTaq® qPCR Master Mix (Promega, Tokyo, Japan) and primers for ABCG2 or GAPDH were used.

The GoTaq^®^ qPCR Master Mix, 2×, was purchased from Promega (Tokyo, Japan). The primer sets for ABCG2 (HA204957-F and HA204957-R) and GAPDH (10000459 and 20000459) were purchased from Takara Bio Inc. (Otsu, Japan).

### 2.6. MTT Assay

Flp-In-293/Mock, Flp-In-293/ABCG2 (WT) and Flp-In-293/ABCG2 (P269S) cells were plated in 96-well plates (Thermo Fisher Scientific) at a density of 5 × 10^3^ cells/well and cultured for 24 h before exposure to different concentrations of mitoxantrone and SN-38 for 72 h. The drug concentrations ranged from 0 (control) to 1000 nM for mitoxantrone and from 0 to 100 nM for SN-38. After drug exposure, cells were incubated with 500 μg/mL MTT for 3 h, lysed with 10% sodium dodecyl sulfate (SDS), and incubated overnight at 37 °C in an atmosphere containing 5% CO_2_. Absorbance was measured at 570 nm using a Thermo Labsystems Multiskan Jax spectrophotometer (Thermo Fisher Scientific) with a reference wavelength of 630 nm to evaluate the amount of formazan metabolized from MTT in each well. Cell viability was calculated as a percentage of the untreated group (control) according to the absorbance at 570 nm with a reference wavelength of 630 nm.

Within each independent experiment, each drug concentration was tested in four technical replicate wells, and the mean viability of the four wells was used for dose–response curve fitting by nonlinear regression using a four-parameter log-logistic model (LL.4) implemented in the drc package in EZR version 1.68, based on R version 4.3.1 (Supplementary Figure S1). The entire experimental procedure was independently conducted five times. The model is represented by the equation f(x) = c + (d − c)/[1 + exp{b(log x − log e)}], where b denotes the slope parameter, c and d represent the lower and upper asymptotes, respectively, and e represents the midpoint between the fitted asymptotes. The upper and lower asymptotes were not fixed but were estimated independently for each dose–response curve. EC_50_ was operationally defined as the drug concentration at which the fitted curve reached 50% cell viability relative to the untreated control and was calculated from each fitted curve rather than being taken directly as the LL.4 midpoint parameter e. Consequently, five independently derived EC_50_ values were obtained for each cell group (*n* = 5). For each fitted curve, the 95% confidence interval (CI) of the EC_50_ estimate was calculated on the logarithmic EC_50_ scale using the delta method and subsequently back-transformed to the original concentration scale. Residual standard error (RSE) was calculated as a measure of residual variation for each fitted curve. Individual EC_50_ estimates, 95% CIs, and RSE values are provided in Supplementary Table S1.

Mitoxantrone and SN-38 were purchased from Wako Pure Chemical Industries, Ltd. (Osaka, Japan) and were graciously provided by Yakult Honsha Co. (Tokyo, Japan), respectively. 3-[4,5-dimethylthiazol-2-yl]-2,5-diphenyltetrazolium bromide (MTT) was obtained from Sigma-Aldrich Co. (St. Louis, MO, USA).

### 2.7. Cell Lysate Preparation for SDS-PAGE

Flp-In-293/Mock, Flp-In-293/ABCG2(WT), and Flp-In-293/ABCG2(P269S) cells were plated in 35 mm dishes (TrueLine, Baton Rouge, LA, USA) at a density of 1 × 10^6^ cells per dish and precultured for 3 days. The cells were harvested with culture medium in 1.5 mL tubes, centrifuged (300× *g*, 5 min, 4 °C), and washed twice with 1 mL PBS(−). The resulting cell pellets were resuspended and lysed in a lysis buffer containing 50 mM Tris-HCl (pH 7.6), 5 mM EDTA (pH 8.0), 120 mM NaCl, 1% Triton X-100, 1 mM DTT, protease inhibitors, and phosphatase inhibitors. The cell lysate samples were homogenized by passing through a 27G needle ten times. After centrifugation of the homogenate at 3000 rpm at 4 °C for 10 min, the supernatants were collected as cell lysates. The protein concentration of the supernatant was determined using bovine serum albumin as a standard by the Bradford method. Following this, 50 μg of the cell lysate was treated with PNGase F for 10 min at 37 °C to deglycosylate ABCG2, thereby improving band resolution for subsequent Western blot analysis. The resulting cell lysates were mixed with SDS-PAGE sample buffer solution containing 10% (*v*/*v*) 2-mercaptoethanol and stored at −30 °C until needed for Western blotting.

PNGase F was obtained from New England Biolabs (Ipswich, MA, USA). SDS-PAGE sample buffer solution containing 10% (*v*/*v*) 2-mercaptoethanol was obtained from Daiichi pure chemicals Co., Ltd. (Tokyo, Japan).

### 2.8. Cell-Surface Protein Biotinylation and Deglycosylation

Flp-In-293/Mock, Flp-In-293/ABCG2(WT), and Flp-In-293/ABCG2(P269S) cells were plated in 60 mm dishes (TrueLine, Baton Rouge, LA, USA) at a density of 2 × 10^6^ cells per dish and precultured for 3 days. After 3 days, the culture medium was removed, and the cells were washed three times with 2 mL of ice-cold PBS(+). The cells were then incubated with 2 mL of Sulfo-NHS-SS-Biotin solution (2 mg/mL in PBS(+)) in each 60 mm dish for 30 min at 4 °C with gentle shaking. After incubation, the solution was removed, and the cells were washed three times with 2 mL of 100 mM glycine in PBS(+) to quench the unreacted biotinylation reagent. The cells were subsequently washed three times with 2 mL of ice-cold PBS(+). During the third wash, the cells were detached in 2 mL of ice-cold PBS(+), collected in 1.5 mL tubes, and centrifuged at 300× *g* for 5 min at 4 °C. After centrifugation, the supernatant was discarded, and the cell pellet was gently loosened by tapping it. The cells were lysed by adding 100 µL of Lysis Buffer B(−), followed by incubation for 30 min with tapping every 10 min. The lysates were then centrifuged at 10,000× *g* for 10 min at 4 °C, and the supernatants were transferred to new 1.5 mL tubes. An aliquot of each cell lysate containing 300 µg of total protein was incubated with 1.8 µL of PNGase F (500,000 U/mL) at 37 °C for 10 min to deglycosylate ABCG2, thereby improving band resolution for subsequent Western blot analysis.

Lysis Buffer B(−) was prepared as a 4× stock solution by mixing 4 mL of Lysis Buffer A, 4 mL of 10% polyethylene glycol mono-p-isooctylphenyl ether (Triton X-100), 400 µL of protease inhibitor cocktail for mammalian cell and tissue extracts (DMSO solution), 400 µL of EDTA-free phosphatase inhibitor cocktail, and 1.2 mL of ultrapure water.

EZ-Link^TM^ Sulfo-NHS-SS Biotin was obtained from Invitrogen (Thermo Fisher Scientific, Waltham, MA, USA). Polyethylene Glycol Mono-p-isooctylphenyl Ether (Triton X-100), protease inhibitor cocktail for mammalian cell and tissue extracts (DMSO solution), and EDTA-free phosphatase inhibitor cocktail were obtained from Nacalai Tesque, Inc. (Kyoto, Japan).

### 2.9. Purification of Biotinylated Proteins

For the purification of biotinylated proteins, 100 µL of NeutrAvidin agarose resin slurry (50 µL packed gel) was mixed with the cell lysate containing 300 µg of total protein in a 2.0 mL tube. The mixture was incubated overnight at 4 °C with rotation using a ROTATOR RT-50 (TAITEC Corporation) to allow the biotinylated proteins to bind to the NeutrAvidin agarose resin. On the following day, the supernatant was discarded after centrifugation at 2500× *g* for 5 min at 4 °C. The resulting resin was then washed by adding 500 µL of Washing Buffer (100 mM potassium phosphate buffer with 150 mM NaCl) and centrifuging at 2000× *g* for 5 min at 4 °C. This washing step was performed three times, and the supernatants were discarded after each centrifugation. After the washing, 25 µL of Elution Buffer, consisting of 2× Laemmli Sample Buffer supplemented with 50 mM DTT, was mixed with the resin and incubated at 37 °C for 30 min to elute the bound proteins from the resin. After incubation, the tubes were centrifuged at 2000× *g* for 2 min at 20 °C, and the supernatants were collected and stored at −80 °C until use.

Pierce^TM^ NeutrAvidin^TM^ Agarose was obtained from Invitrogen (Thermo Fisher Scientific, Waltham, MA, USA).

### 2.10. Evaluation of Expression Levels of ABCG2

For each cell group, lysates from three independent experiments were pooled by combining equal protein amounts (5 μg from each experiment). The pooled sample was loaded into three separate lanes on the same 7.5% polyacrylamide gel and fractionated by SDS-PAGE, followed by transfer to a nitrocellulose membrane (GE Healthcare UK Ltd., Bucks, UK). Western blotting was performed after the blocking process, in which the membranes were immersed in TBST (50 mM Tris-HCl, 150 mM NaCl, and 0.05% (*v*/*v*) Tween 20) containing skimmed milk powder at 5% (*w*/*v*) at room temperature for more than 1 h and then at 4 °C overnight. After this procedure, the membranes were rinsed with TTBS and exposed to a 1:1000-diluted monoclonal anti-ABCG2 antibody (BXP-21; ALEXIS Co., Lausen, Switzerland), Anti-N-Cadherin (BD Transduction Laboratories^TM^), or anti-GAPDH antibody (anti-GAPDH-Clone 6C5 mouse monoclonal, IgG2b; American Research Products, Inc., Waltham, MA, USA) in TBST containing skimmed milk powder at 5% (*w*/*v*) with gentle shaking for 1 h at room temperature. After primary antibody treatment, the membranes were rinsed with TBST and exposed to 1:1000-diluted HRP-conjugated anti-mouse IgG antibodies (Cell Signaling Technology, Inc., Danvers, MA, USA) in 5% (*w*/*v*) skim milk powder-containing TBST with gentle shaking for 1 h at room temperature. Blots were visualized using Western Lightning Chemiluminescence Reagent Plus (PerkinElmer Life and Analytical Sciences, Boston, MA, USA) and detected using WSE-6100 LuminoGraph I (Atto Corp., Tokyo, Japan). Signal intensities of ABCG2, N-cadherin, and GAPDH were quantified using ImageJ software (version 1.49v, Wayne Rasband, Bethesda, MD, USA), with densitometric measurements obtained separately from the three lanes of each pooled sample. For quantitative analysis, GAPDH was used as a loading reference for total ABCG2 protein levels, whereas N-cadherin was used as a plasma membrane-associated reference protein for normalization of ABCG2 signals in the biotinylated protein fraction.

### 2.11. Statistical Analysis

All statistical analyses were performed using EZR version 1.68 (Saitama Medical Center, Jichi Medical University, Saitama, Japan) [31], a graphical user interface for R (version 4.3.1). For the MTT assays, the experimental unit was an independently performed experiment, and five independently derived EC_50_ values were analyzed for each cell group (*n* = 5). Technical replicate wells were not considered independent observations. Because the normality assumption was not satisfied for the re-estimated EC_50_ values, differences among the three cell groups were evaluated using the Kruskal–Wallis test. Pairwise comparisons were performed using two-sided exact Wilcoxon rank-sum tests with Holm adjustment for multiple comparisons. For descriptive presentation, the EC_50_ values were reported on the original concentration scale as mean ± S.D.

For the qPCR and Western blot densitometric analyses, one-way ANOVA followed by Tukey’s HSD test was used, as indicated in the corresponding figure legends. For densitometric analysis of total ABCG2 protein and ABCG2 abundance in the biotinylated protein fraction, the statistical comparisons were based on measurements obtained from three separate lanes loaded with the same pooled lysate sample. These measurements were treated as technical replicates and were not used to infer biological variability among independently prepared samples. Statistical significance was defined as *p* < 0.05, unless otherwise indicated in the corresponding figure legend.

## 3. Results

### 3.1. Expression Levels of ABCG2 mRNA and Protein in Cells Expressing ABCG2 (P269S)

**Figure 2:**
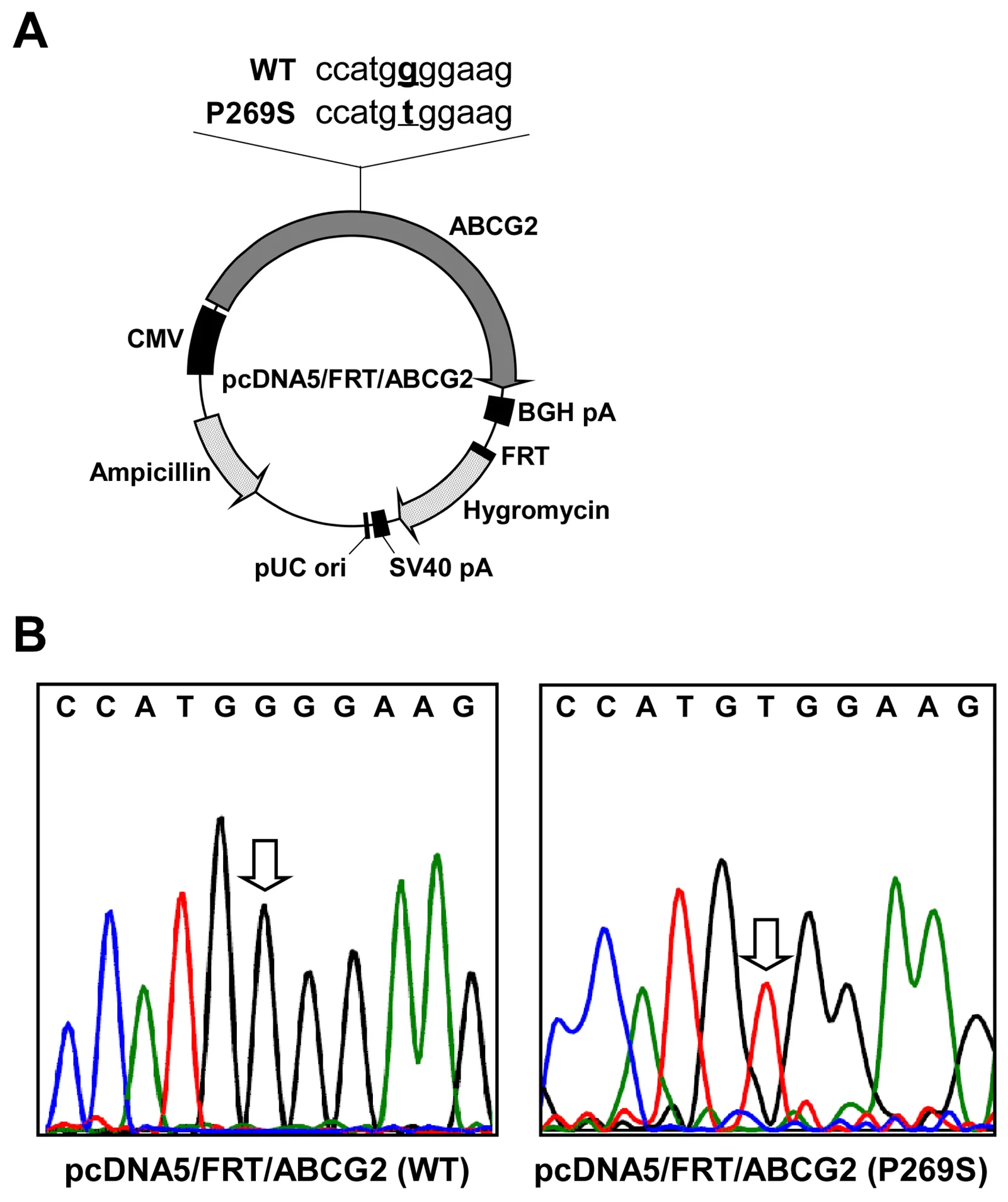
Schematic illustration of the pcDNA5/FRT/ABCG2 vector (**A**) and electropherogram of DNA Sequencing (**B**). (**A**) Partial cDNA sequences at positions 800 to 810 in *ABCG2 (WT)* and (*P269S*) cDNA are indicated. The base at position 805 is indicated by a bold underline. ABCG2, *ABCG2* cDNA; BGH, bovine growth hormone; CMV, cytomegalovirus; pA, polyadenylation sequence; pUC ori, pUC vector origin of replication; SV40, simian virus 40. (**B**) Electropherograms confirming the C>T substitution at position 805 introduced into *ABCG2(WT)* cDNA by site-directed mutagenesis. The nucleotide at position 805 is indicated by the arrows.

To quantitatively evaluate the effect of SNP rs34678167 (c.805C>T, P269S) on ABCG2 protein expression and cellular resistance to mitoxantrone and SN-38, we established Flp-In-293 cells expressing ABCG2 (P269S) (Figure 1 and Figure 2). Total RNA was extracted from Flp-In-293/Mock, Flp-In-293/ABCG2(WT), and Flp-In-293/ABCG2(P269S) cells, and RNA quality was evaluated by comparing A260/A280 ratios and qPCR threshold cycles across samples. Because RNA quality was comparable among samples, ABCG2 mRNA levels were normalized to GAPDH mRNA levels. The detection of robust ABCG2(P269S) mRNA expression, together with hygromycin-based selection using the Flp-In™ system, was consistent with successful establishment of Flp-In-293/ABCG2(P269S) cells.

**Figure 3:**
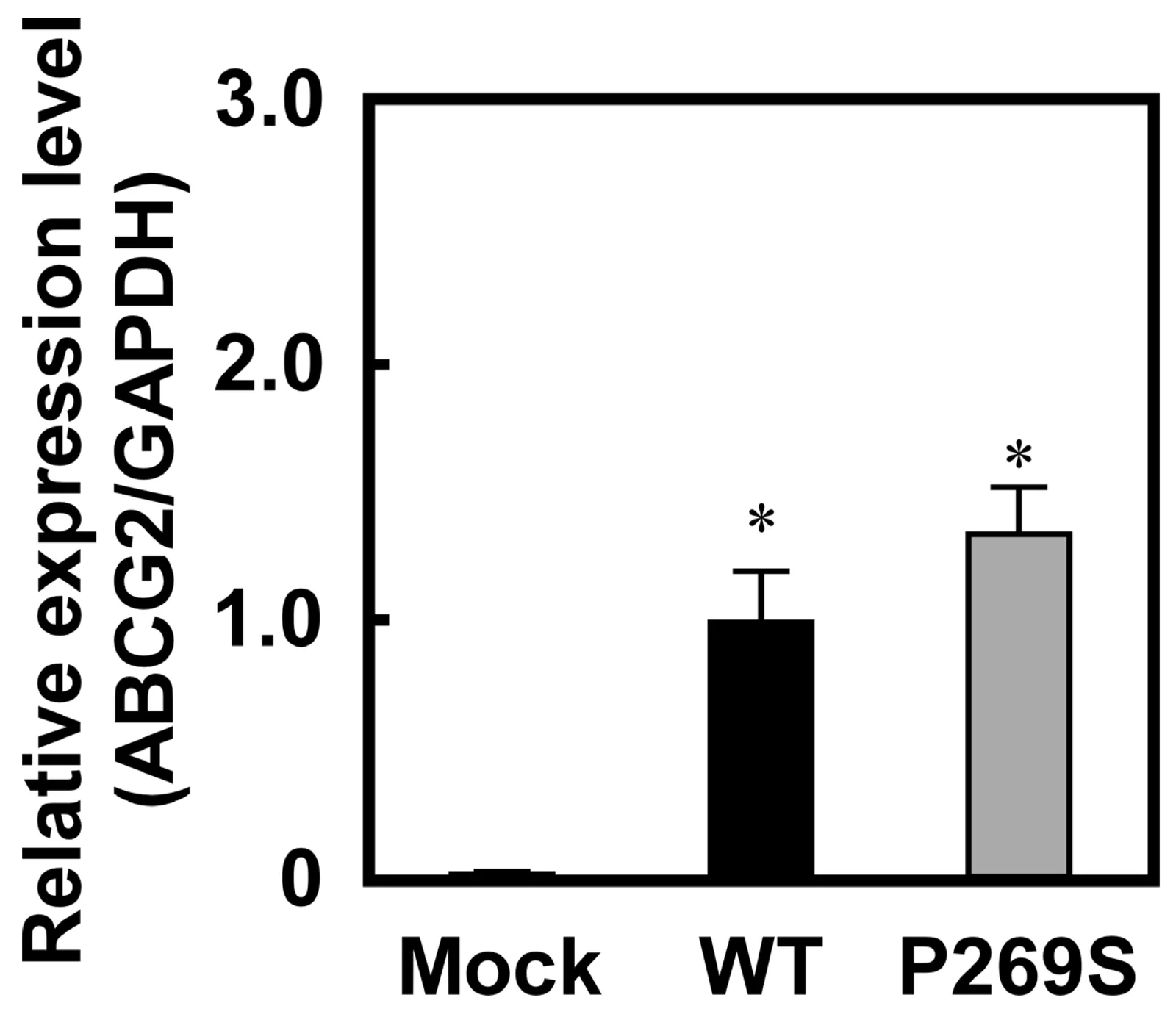
mRNA levels of ABCG2 in cells established using the Flp-In™ system. The levels of *ABCG2* and *GAPDH* mRNA were measured using qPCR with specific primer sets for *ABCG2* and *GAPDH*, as described in Materials and Methods. Data are calculated as ratios relative to the *GAPDH* mRNA levels in the cells and normalized to the ratio of ABCG2/GAPDH. Data are expressed as mean values ± S.D. (*n* = 5). Statistical analyses were performed using one-way ANOVA and Tukey’s HSD test (* *p* < 0.01 compared to the Mock group).

The results of qPCR showed that ABCG2 mRNA levels in ABCG2 cDNA-transfected cells [Flp-In-293/ABCG2 (WT) and Flp-In-293/ABCG2 (P269S) cells] were more than 100 times higher than those in Flp-In-293/Mock cells (Figure 3). ABCG2 mRNA levels in Flp-In-293/ABCG2 (P269S) cells were comparable to those in Flp-In-293/ABCG2 (WT) cells, supporting comparable transgene expression between the two ABCG2-expressing cell lines under the Flp-In™ system.

**Figure 4:**
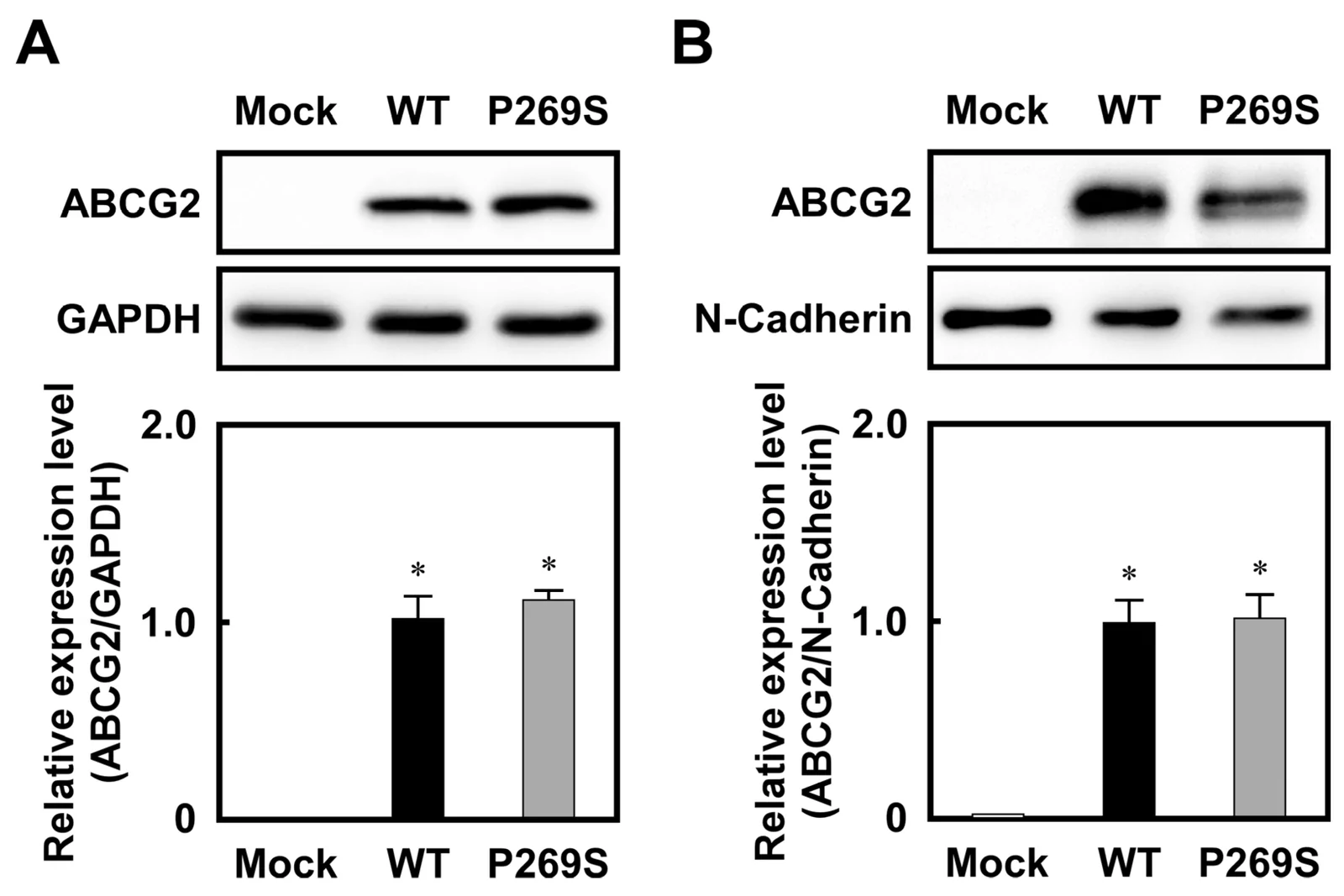
Total ABCG2 protein and ABCG2 abundance in the biotinylated protein fraction in cells established using the Flp-In™ system. (**A**) Total ABCG2 protein expression levels in Flp-In-293/Mock, Flp-In-293/ABCG2 (WT), and Flp-In-293/ABCG2 (P269S) cells were determined by Western blotting. ABCG2 expression levels were normalized to those of GAPDH. (**B**) ABCG2 abundance in the biotinylated protein fraction was determined by Western blotting (original images can be found at Figure S2) and normalized to N-cadherin, a plasma membrane-associated reference protein. Data are expressed as the mean ± S.D. of three technical replicate measurements from the same pooled sample (*n* = 3 technical replicates). Statistical analyses were performed using one-way ANOVA followed by Tukey’s HSD test (* *p* < 0.05 compared with the Mock group). Statistical comparisons reflect technical replicate measurements and do not represent biological variability among independently prepared samples.

Western blotting was performed to determine ABCG2 protein expression levels in Flp-In-293/ABCG2 (WT) and Flp-In-293/ABCG2 (P269S) cells. PNGase F treatment was used to reduce the influence of glycosylation on the electrophoretic appearance of ABCG2 and to improve band resolution for quantitative analysis. As shown in Figure 4A, the ABCG2 signal intensity was higher in Flp-In-293/ABCG2(WT) and Flp-In-293/ABCG2(P269S) cells than in Flp-In-293/Mock cells under the analytical conditions used. The normalized ABCG2 signal intensities were comparable between Flp-In-293/ABCG2(P269S) and Flp-In-293/ABCG2(WT) cells.

To further examine whether the P269S variant affected cell-surface abundance of ABCG2, cell surface proteins in Flp-In-293/Mock, Flp-In-293/ABCG2 (WT), and Flp-In-293/ABCG2 (P269S) cells were biotinylated and purified using avidin-binding resin. As shown in Figure 4B, ABCG2 was readily detected in the biotinylated protein fraction from Flp-In-293/ABCG2(WT) and Flp-In-293/ABCG2(P269S) cells, whereas only minimal or background-level signal was observed in Flp-In-293/Mock cells. Quantitative analysis after normalization to the plasma membrane-associated reference protein N-cadherin showed comparable ABCG2 signal intensities in the biotinylated protein fraction between Flp-In-293/ABCG2(WT) and Flp-In-293/ABCG2(P269S) cells.

### 3.2. Evaluation of Cellular Resistance to Mitoxantrone and SN-38 in Cells Expressing ABCG2 (P269S)

To investigate the impact of rs34678167 (c.805C>T, P269S) on cellular resistance to mitoxantrone and SN-38, we performed an MTT assay to assess cellular responses to these drugs. The cells were treated with varying concentrations of mitoxantrone or SN-38, and their EC_50_ values were determined by nonlinear regression analysis of five independent experiments using a four-parameter log-logistic model (Supplementary Figure S1 and Table S1). The mean EC_50_ values for mitoxantrone were 6.5 ± 5.3, 19 ± 4.7, and 57 ± 12 nM in Flp-In-293/Mock, Flp-In-293/ABCG2(WT), and Flp-In-293/ABCG2(P269S) cells, respectively (Table 1). For SN-38, the corresponding EC_50_ values were 2.9 ± 1.2, 17 ± 2.0, and 60 ± 7.0 nM, respectively (Table 1). In all 30 dose–response datasets, cell viability decreased below 50% of the untreated control within the tested concentration range; thus, no extrapolation beyond the tested concentrations was required to estimate EC_50_ values.

**Figure 5:**
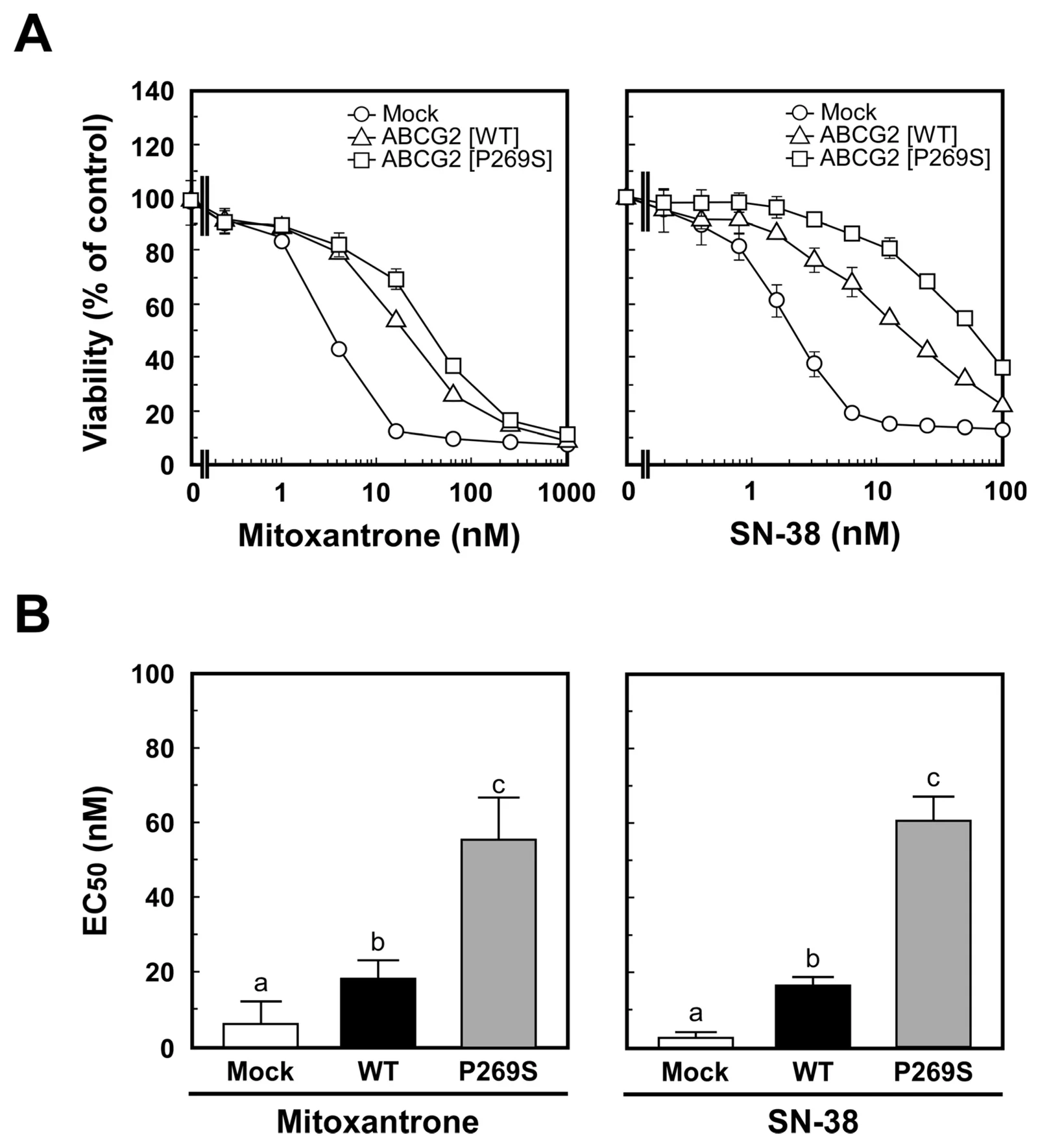
Cellular responses to mitoxantrone and SN-38 in cells established using the Flp-In™ system. (**A**) Representative dose–response curves from one of five independently performed experiments are shown for each drug. Each point represents the mean ± S.D. of four technical replicate wells within the representative experiment (*n* = 4 technical replicates). (**B**) Individual EC_50_ values obtained from each of five independently performed experiments, for which the corresponding dose–response curves are shown in Supplementary Figure S1, are shown together with the mean ± S.D. (*n* = 5 independent experiments). Statistical comparisons among cell groups were performed using the Kruskal–Wallis test followed by pairwise two-sided exact Wilcoxon rank-sum tests with Holm adjustment for multiple comparisons. Different letters indicate significant differences between groups (Holm-adjusted *p* < 0.05). Individual EC_50_ estimates, 95% CIs, and RSE values are provided in Supplementary Table S1.

Consistent with the results of previous studies [12,21,27], Flp-In-293/ABCG2(WT) cells exhibited increased resistance to mitoxantrone and SN-38 compared with Flp-In-293/Mock cells. Based on the unrounded mean EC_50_ values, the EC_50_ values for mitoxantrone and SN-38 were approximately 3.0- and 5.8-fold higher, respectively, in Flp-In-293/ABCG2(WT) cells than in Flp-In-293/Mock cells (Figure 5 and Table 1). Flp-In-293/ABCG2(P269S) cells exhibited even higher mean EC_50_ values than Flp-In-293/ABCG2(WT) cells, by approximately 3.0-fold for mitoxantrone and 3.5-fold for SN-38 (Figure 5 and Table 1). The Kruskal–Wallis test showed significant differences in EC_50_ values among the three cell groups for both mitoxantrone (χ^2^ = 12.02, df = 2, *p* = 0.00245) and SN-38 (χ^2^ = 12.50, df = 2, *p* = 0.00193). Because significant overall group differences were detected, pairwise exact Wilcoxon rank-sum tests with Holm adjustment for multiple comparisons were subsequently performed. These analyses showed significant differences for all three pairwise comparisons for both drugs. In particular, Flp-In-293/ABCG2(P269S) cells had significantly higher EC_50_ values than Flp-In-293/ABCG2(WT) cells for both mitoxantrone and SN-38 (exact *p* = 0.00794; Holm-adjusted *p* = 0.0238 for each drug). The Mock-versus-WT and Mock-versus-P269S comparisons were also significant for both drugs (Holm-adjusted *p* = 0.0238 for each comparison).

## 4. Discussion

### 4.1. Generation of Human ABCG2 (P269S)-Expressing Cells Using the Flp-In™ System

The emergence of drug resistance in cancer cells and its inter-individual variability have been attributed, in part, to the overexpression of ABC transporters and the presence of specific single-nucleotide polymorphisms (SNPs) in their genes [12,21,27,30]. Our previous studies demonstrated that several non-synonymous SNPs in *ABCG2*, such as rs2231137 (V12M), rs2231142 (Q141K), rs1061018 (F208S), rs3116448 (S248P), rs1354553769 (S441N), and rs192169063 (F489L), can influence ABCG2-mediated drug resistance in cells expressing ABCG2 [27]. In this study, we used the Flp-In™ system to generate cells expressing the rs34678167 (P269S) variant of ABCG2. This system enables site-specific integration of a single copy of cDNA at the FRT site in the telomeric region of chromosome 12 in Flp-In-293 cells [27,32], providing a controlled platform for quantitatively assessing the functional consequences of individual SNPs on drug resistance. Although we did not directly confirm the genomic integration site and copy number of the cDNA in Flp-In-293/ABCG2 (P269S) cells, the comparable ABCG2 mRNA levels between ABCG2(P269S)- and ABCG2(WT)-expressing cells (Figure 3) supported comparable expression under the Flp-In™ system.

### 4.2. Cellular Resistance to Mitoxantrone and SN-38 in ABCG2(P269S)-Expressing Cells

ABCG2 has been shown to mediate the efflux of a broad range of substrates, including anticancer drugs such as camptothecin and its analogs [7,8,12,16], epidermal growth factor receptor tyrosine kinase inhibitors [18,19], mitoxantrone [8,20,21], and methotrexate [22,23], as well as endogenous compounds like dehydroepiandrosterone sulfate [9], estrone-3-sulfate [9,10], lumichrome [11], porphyrins [12,13,14], and uric acid [15]. As shown in Figure 5 and Table 1, our results confirmed that Flp-In-293/ABCG2 (WT) cells exhibited increased resistance to mitoxantrone and SN-38 compared with Flp-In-293/Mock cells, consistent with the findings of previous studies [7,8,12,16,17,20,21,27]. Importantly, Flp-In-293/ABCG2(P269S) cells exhibited significantly higher EC_50_ values for both drugs than Flp-In-293/ABCG2(WT) cells (Figure 5 and Table 1). The increased resistance in ABCG2(WT)-expressing cells compared with Mock cells, together with the further increase in resistance associated with P269S in the same Flp-In-293 cellular background, supports the involvement of ABCG2 expression and its P269S variant in the observed cellular resistance phenotype. However, these findings alone do not establish that the differences in cellular resistance are attributable exclusively to ABCG2-mediated drug efflux.

As outlined in Section 4.1, the Flp-In™ system provides a uniform cellular background suitable for evaluating the effects of single-nucleotide substitutions in ABCG2 [27]. In Flp-In-293 cells stably expressing either ABCG2(WT) or ABCG2(P269S), ABCG2 mRNA levels, total ABCG2 protein expression, and ABCG2 abundance in the biotinylated protein fraction were comparable between the two cell lines (Figure 3 and Figure 4). Despite these comparable expression profiles, Flp-In-293/ABCG2(P269S) cells exhibited increased resistance to mitoxantrone and SN-38 compared with Flp-In-293/ABCG2(WT) cells. However, because contamination of the biotinylated fraction by intracellular proteins such as GAPDH was not assessed in the present study, the current data should be interpreted as showing no detectable difference in ABCG2 abundance in the biotinylated protein fraction rather than definitively establishing equivalent cell-surface localization between WT- and P269S-expressing cells. In addition, we cannot exclude the possibility that the introduction and overexpression of ABCG2 induced the expression of other drug-efflux ABC transporters, which may have contributed to the observed cellular resistance phenotype. Despite these limitations, the present findings suggest that the increased resistance phenotype cannot be explained simply by increased ABCG2 abundance at the whole-cell level or in the biotinylated protein fraction. They also raise the possibility that the 805C>T substitution (rs34678167, P269S) alters substrate-dependent functional properties of ABCG2 without producing detectable changes in its overall expression level or abundance in the biotinylated protein fraction.

### 4.3. Implications of P269S (rs34678167, c.805C>T) for Substrate-Dependent ABCG2 Function

Several studies have assessed the transport activity of ABCG2(P269S) using membrane vesicles. Kondo et al. [9] reported that P269S exhibited transport activities comparable to those of ABCG2(WT) for estrone-3-sulfate, dehydroepiandrosterone sulfate, methotrexate, and p-aminohippurate, using membrane vesicles prepared from adenovirus-infected HEK293 cells. Similarly, Higashino et al. [28] found no significant alteration in the urate transport activity of P269S using membrane vesicles prepared from transiently transfected human embryonic kidney-derived cells. In contrast, Lee et al. [29] reported an approximately 35–40% reduction in the transport of estrone-3-sulfate and methotrexate by P269S using membrane vesicles prepared from baculovirus-infected Sf9 cells, despite comparable protein expression levels between P269S and WT. Lee et al. [29] also found no difference in ATPase activity between P269S and WT and suggested that differences in expression systems and membrane lipid environments between Sf9 and mammalian cells could contribute to the discrepancy with the results obtained in HEK293 cells.

The present study differs from these previous studies in several important respects. Whereas the previous studies directly measured ATP-dependent transport of individual substrates in isolated membrane vesicles [9,28,29], we evaluated cellular responses to mitoxantrone and SN-38 in intact Flp-In-293 cells using a 72 h MTT assay. Thus, both the substrates examined and the functional endpoints differ substantially between the studies. In addition, the Flp-In™ system uses stable, site-specific expression in a mammalian cellular background, whereas the previous transport studies used adenoviral expression in HEK293 cells [9], transient expression in human embryonic kidney-derived cells [28], or baculoviral expression in Sf9 insect cells [29]. These differences in substrates, cellular versus vesicular environments, protein expression systems, membrane composition, assay conditions, and functional endpoints may contribute to the different P269S phenotypes observed among studies. Accordingly, the present findings do not necessarily contradict the previous transport studies but rather represent a distinct cellular phenotype assessed using different substrates and a different functional endpoint. Because these factors were not directly compared in the present study, their contributions remain hypothetical. Furthermore, because only mitoxantrone and SN-38 were examined, the observed phenotype should not be interpreted as a generalized gain of ABCG2 function, and the effects of P269S on other ABCG2 substrates remain unknown.

The present study does not directly define the molecular mechanism underlying the P269S-associated cellular resistance phenotype. ABCG2 transport activity was not directly measured, and ABCG2 inhibition or knockout/rescue experiments were not performed; therefore, contributions from ABCG2-independent cellular factors cannot be excluded. In particular, we cannot exclude the possibility that the introduction of ABCG2(WT) or ABCG2(P269S) altered the expression of other drug-efflux ABC transporters, which may have contributed to the observed differences in cellular resistance. Because the MTT assay reflects cellular metabolic activity rather than transporter function, differences in proliferation, metabolic state, stress tolerance, apoptosis, or other determinants of drug sensitivity may also contribute to the observed phenotype. In addition, intracellular drug accumulation, substrate efflux, ATPase activity, conformational changes, oligomerization state, and protein–protein interactions were not examined. These limitations leave open the possibility that P269S affects substrate recognition, catalytic turnover, coupling between ATP hydrolysis and substrate transport, or the local membrane environment of ABCG2, as suggested for other ABCG2 substitutions, including V12M, Q141K, F208S, S248P, F431L, S441N, and F489L [12,30]. However, these mechanisms remain hypothetical and require further investigation. Therefore, the observed increase in cellular resistance should not be interpreted as direct evidence of enhanced ABCG2 transport activity.

The present findings were obtained in a single engineered Flp-In-293 cellular background, in which ABCG2 was expressed at levels substantially higher than in Mock cells. Additional cellular backgrounds, endogenous genome-edited models, and independently generated cell populations were not examined. Therefore, the generalizability of the present findings to endogenous or physiological ABCG2 expression conditions remains to be established. Accordingly, the present study does not establish a clinical pharmacogenomic effect of rs34678167. Despite these limitations, the present study demonstrates that, within a controlled Flp-In-293 cellular background, the P269S variant is associated with significantly increased cellular resistance to mitoxantrone and SN-38 compared with ABCG2(WT), without detectable differences in ABCG2 mRNA levels, total ABCG2 protein expression, or ABCG2 abundance in the biotinylated protein fraction. These findings establish a distinct P269S-associated cellular resistance phenotype that cannot be explained simply by increased ABCG2 abundance and provide a basis for further investigation of the substrate-dependent functional consequences and molecular mechanisms associated with this variant.

## 5. Conclusions

In this study, we employed a quantitative and site-specific expression system to evaluate the impact of the SNP rs34678167 (c.805C>T, P269S) in the human ABCG2 gene on cellular resistance to mitoxantrone and SN-38. Consistent with our previous studies [12,21,27,30], the Flp-In™ system enabled the generation of cells expressing ABCG2(P269S) under conditions comparable to those expressing ABCG2(WT), providing a controlled cellular background for comparison of the two ABCG2 forms. The genomic integration site and cDNA copy number were not directly confirmed in the present study, and the findings are limited to this engineered Flp-In-293 expression system.

Our results demonstrated that cells expressing ABCG2(P269S) showed increased cellular resistance to mitoxantrone and SN-38 compared with cells expressing ABCG2(WT) (Figure 5), despite no detectable differences in ABCG2 mRNA levels (Figure 3), total ABCG2 protein levels (Figure 4A), or ABCG2 abundance in the biotinylated protein fraction (Figure 4B) under the present experimental conditions. Although our findings do not establish equivalent protein folding, maturation, oligomerization, or functional state between ABCG2(WT) and ABCG2(P269S), the increased cellular resistance associated with P269S was not accompanied by detectable differences in ABCG2 abundance. Because only mitoxantrone and SN-38 were examined in the present study, the present findings should not be interpreted as evidence of a generalized gain of ABCG2 function. Nevertheless, the present study provides a rationale for further investigation of the substrate specificity and molecular basis of the P269S-associated phenotype.

## Figures and Tables

**Table 1:** Anticancer drug resistance profiles (EC_50_) of the cells.

Compounds	EC_50_ (nM)								
	Mock Cells			ABCG2 (WT) Cells			ABCG2 (P269S) Cells		
Mitoxantrone	6.5	±	5.3 ^a^	19	±	4.7 ^b^	57	±	12 ^c^
SN-38	2.9	±	1.2 ^a^	17	±	2.0 ^b^	60	±	7.0 ^c^

SN-38, 7-ethyl-10-hydroxycamptothecin. EC_50_ values were obtained by nonlinear regression using a four-parameter log-logistic model with the upper and lower asymptotes estimated independently for each curve without fixing their values. EC_50_ was defined as the drug concentration corresponding to 50% cell viability relative to the untreated control, rather than the LL.4 midpoint parameter e. Data are expressed as mean ± S.D. from five independently performed experiments (*n* = 5; Supplementary Figure S1). Each independent experiment yielded one EC_50_ value; technical replicate wells were not treated as independent observations. Statistical comparisons among cell groups were performed using the Kruskal–Wallis test followed by pairwise two-sided exact Wilcoxon rank-sum tests with Holm adjustment for multiple comparisons. Within each row, values with different superscript letters are significantly different (Holm-adjusted *p* < 0.05). Individual EC_50_ estimates, 95% CIs, and RSE values for each fitted curve are provided in Supplementary Table S1.

## Data Availability

The raw data supporting the conclusions of this article will be made available by the authors on request.

## Supplementary Materials

The following supporting information can be downloaded at: https://www.mdpi.com/article/10.3390/biom16101486/s1, Figure S1: Dose–response curves for mitoxantrone and SN-38 in Flp-In-293/Mock, Flp-In-293/ABCG2(WT), and Flp-In-293/ABCG2(P269S) cells. (A–C) Dose–response curves for mitoxantrone in Flp-In-293/Mock (A), Flp-In-293/ABCG2(WT) (B), and Flp-In-293/ABCG2(P269S) (C) cells. (D–F) Dose–response curves for SN-38 in Flp-In-293/Mock (D), Flp-In-293/ABCG2(WT) (E), and Flp-In-293/ABCG2(P269S) (F) cells. Each panel shows the results of five independently performed experiments (Ex 1–5). At each drug concentration, four technical replicate wells were measured, and data are presented as the mean ± SD of the four wells within the corresponding independent experiment. Cell viability is expressed as a percentage of the untreated control. Dose–response data from each independent experiment were fitted separately by nonlinear regression using a four-parameter log-logistic model (LL.4). Each independent experiment yielded one EC_50_ estimate. Individual EC_50_ estimates, 95% confidence intervals, and residual standard error values are provided in Supplementary Table S1; Figure S2: Western Blot Original image; Table S1: EC_50_ estimates, 95% confidence intervals, and residual standard errors for individual mitoxantrone and SN-38 dose–response curve fits.
