# Supplementary material for: A Human ABCG2 SNP Variant, P269S (rs34678167, c.805C>T), Increases Cellular Resistance to Mitoxantrone and SN-38 Without Detectable Differences in ABCG2 Abundance in Total and Biotinylated Protein Fractions
Source: Biomolecules. 2026 Oct 13;16(10):1486. doi: 10.3390/biom16101486 (PMC13702490; doi:10.3390/biom16101486)

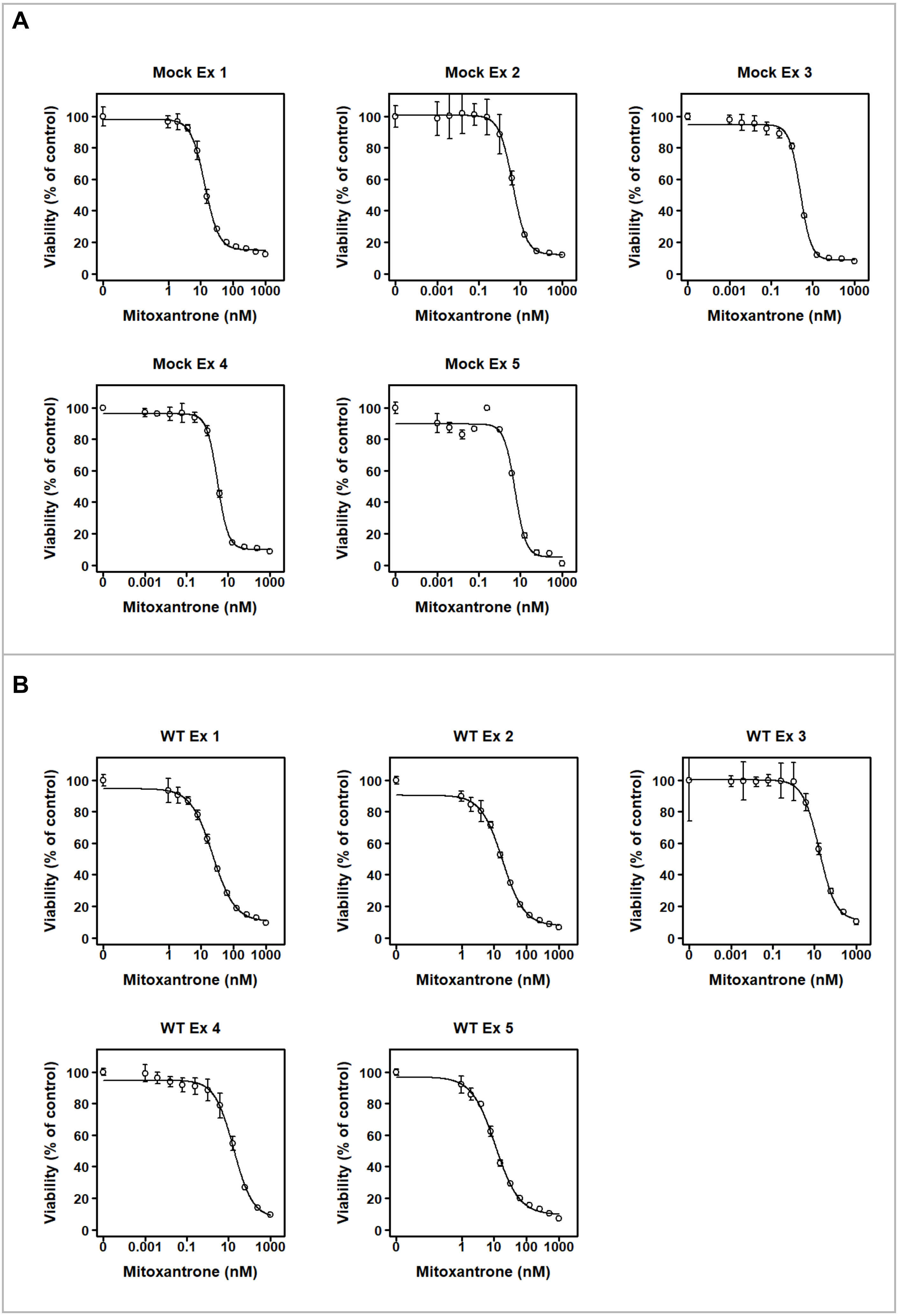
A
Mock Ex 1
Mock Ex 2
Mock Ex 3
Mock Ex 4
Mock Ex 5
B
WT Ex 1
WT Ex 2
WT Ex 3
WT Ex 4
WT Ex 5
Viability (% of control)
Mitoxantrone (nM)
0
20
40
60
80
100
0.001
0.1
1
10
100
1000

C

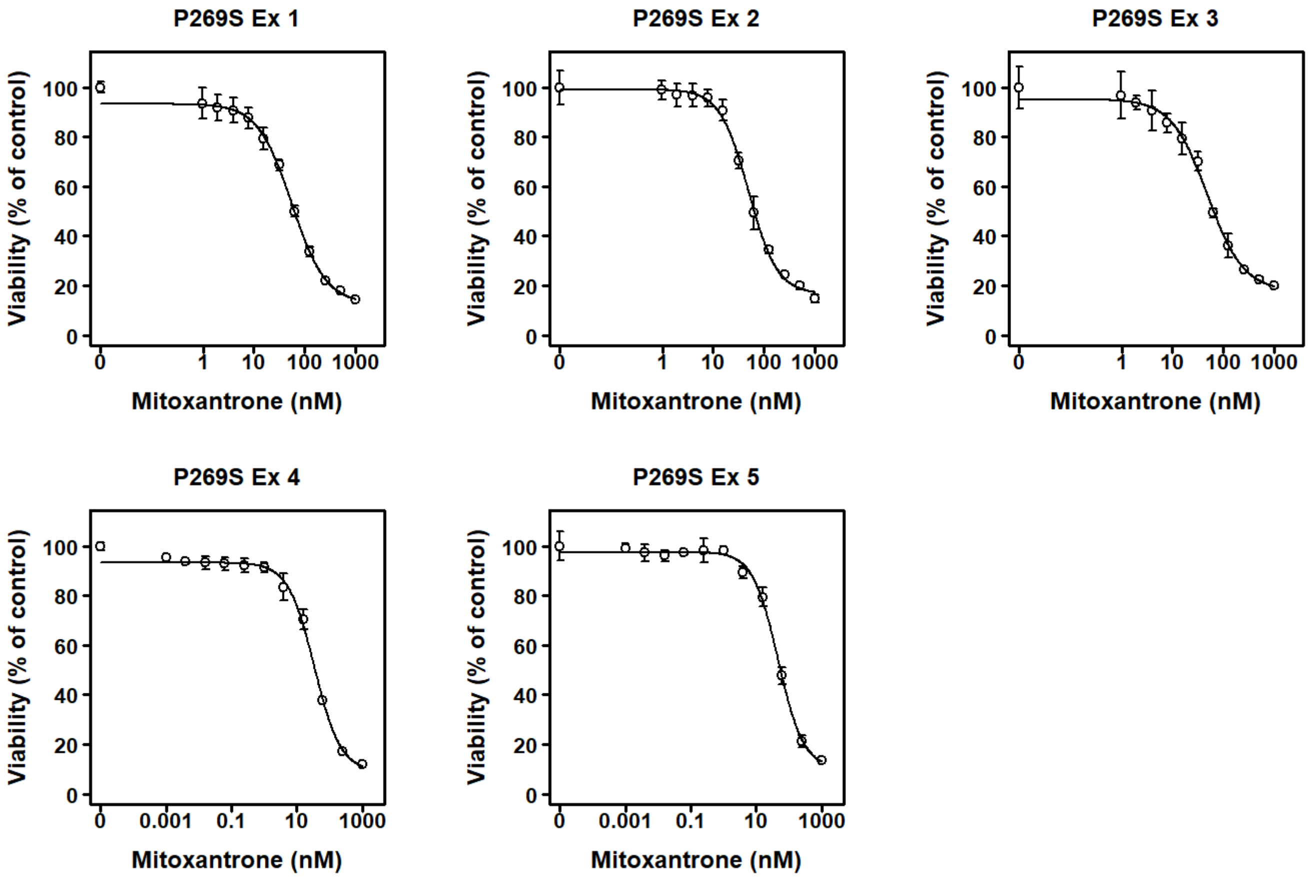
P269S Ex 1
P269S Ex 2
P269S Ex 3
P269S Ex 4
P269S Ex 5
Viability (% of control)
100
80
60
40
20
0
0
1
10
100
1000
0.001
0.1
Mitoxantrone (nM)

D

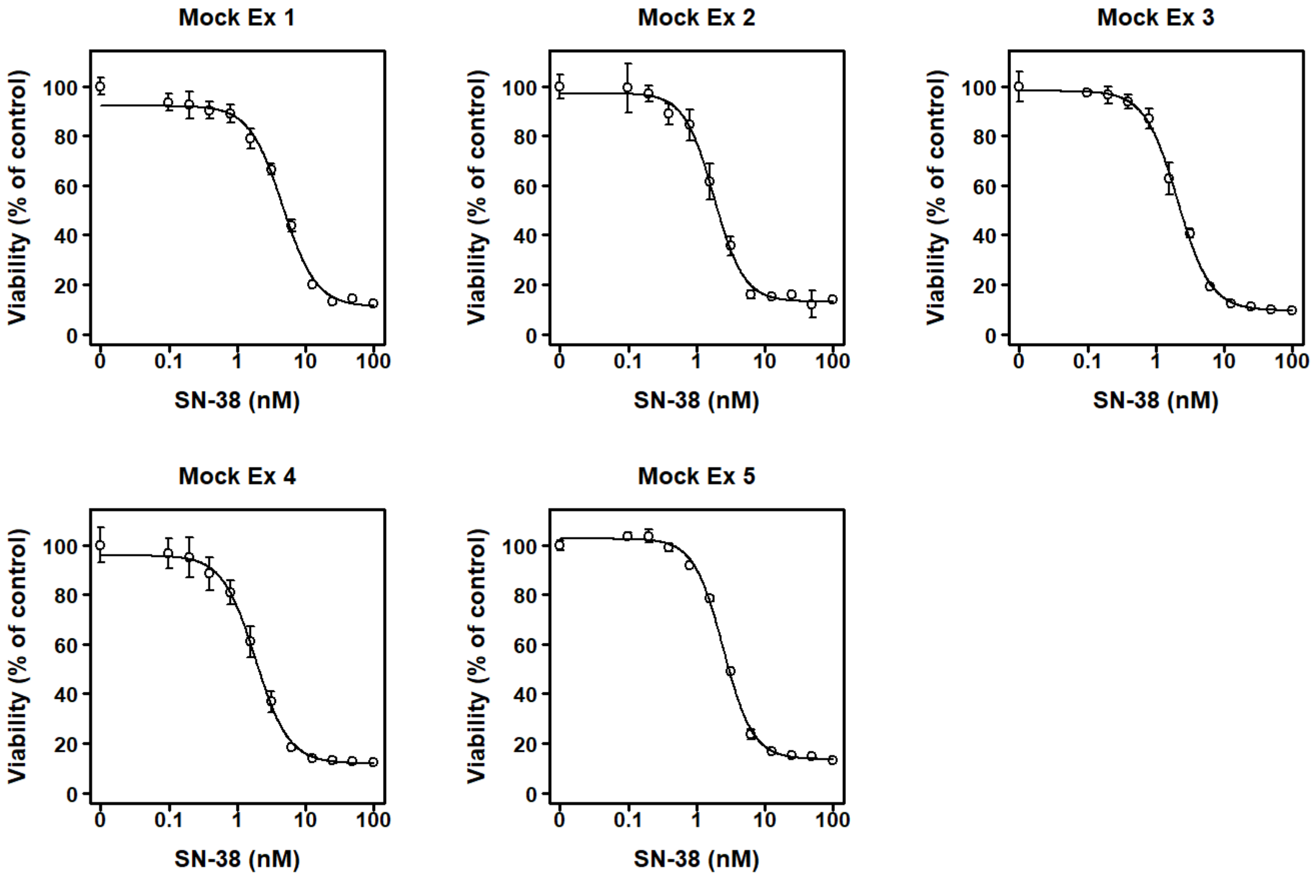
Mock Ex 1
Mock Ex 2
Mock Ex 3
Mock Ex 4
Mock Ex 5
Viability (% of control)
100
80
60
40
20
0
0
0.1
1
10
100
SN-38 (nM)

E

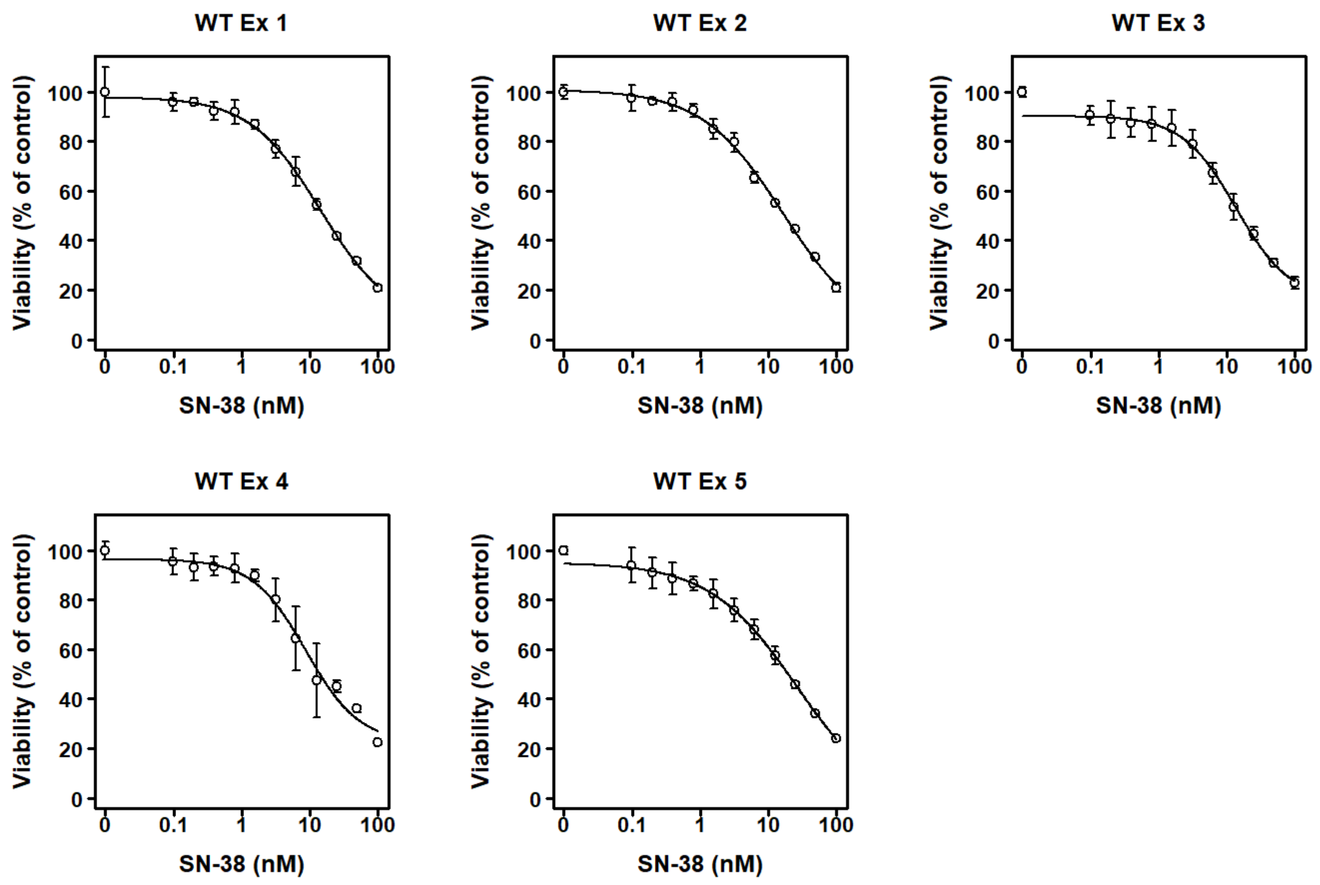
WT Ex 1
WT Ex 2
WT Ex 3
WT Ex 4
WT Ex 5
Viability (% of control)
SN-38 (nM)
0
20
40
60
80
100
0.1
1
10

F

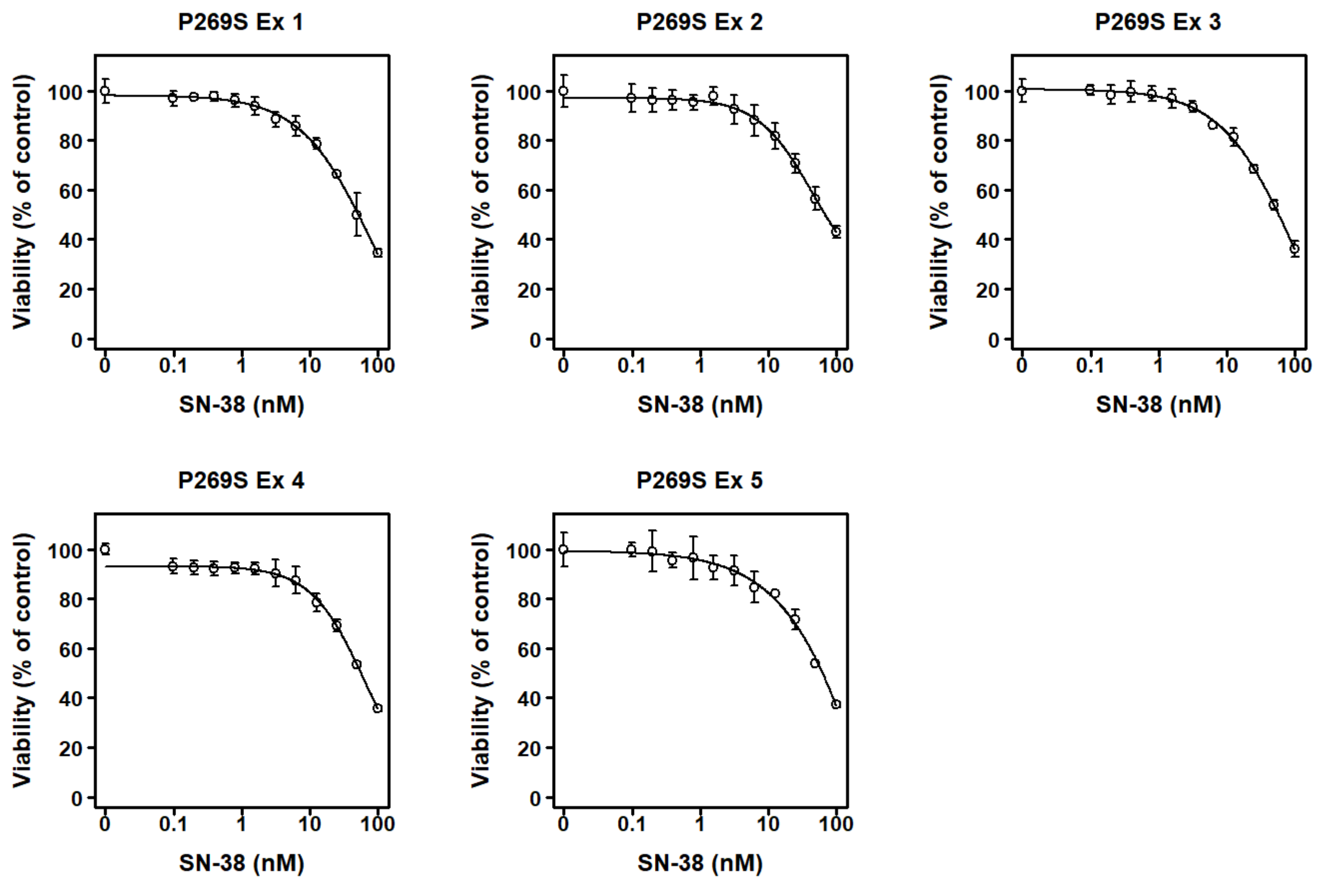
P269S Ex 1
P269S Ex 2
P269S Ex 3
P269S Ex 4
P269S Ex 5
Viability (% of control)
SN-38 (nM)
0
20
40
60
80
100
0.1
1
10

Supplementary Figure S1. Dose-response curves for mitoxantrone and SN-38 in Flp-In-293/Mock, Flp-In-293/ABCG2(WT), and Flp-In-293/ABCG2(P269S) cells. (A-C) Dose-response curves for mitoxantrone in Flp-In-293/Mock (A), Flp-In-293/ABCG2(WT) (B), and Flp-In-293/ABCG2(P269S) (C) cells. (D-F) Dose-response curves for SN-38 in Flp-In-293/Mock (D), Flp-In-293/ABCG2(WT) (E), and Flp-In-293/ABCG2(P269S) (F) cells. Each panel shows the results of five independently performed experiments (Ex 1-5). At each drug concentration, four technical replicate wells were measured, and data are presented as the mean ± SD of the four wells within the corresponding independent experiment. Cell viability is expressed as a percentage of the untreated control. Dose-response data from each independent experiment were fitted separately by nonlinear regression using a four-parameter log-logistic model (LL.4). Each independent experiment yielded one EC_{50} estimate. Individual EC_{50} estimates, 95% confidence intervals, and residual standard error values are provided in Supplementary Table S1.

Table S1. EC_{50} estimates, 95% confidence intervals, and residual standard errors for individual mitoxantrone and SN-38 dose-response curve fits.

	Mock cells			ABCG2(WT) cells			ABCG2(P269S) cells		
	**EC_{50} (nM)**	**95% CI (nM)**	**RSE**	**EC_{50} (nM)**	**95% CI (nM)**	**RSE**	**EC_{50} (nM)**	**95% CI (nM)**	**RSE**
**Mitoxantrone**									
Ex 1	16	15–17	1.5	25	24–26	0.85	63	61–65	0.78
Ex 2	5.4	5.0–5.9	1.4	18	17–19	1.3	64	58–71	2.2
Ex 3	2.7	2.4–3.0	2.8	22	19–24	1.6	65	58–72	1.9
Ex 4	3.4	3.2–3.6	1.0	19	16–22	2.3	37	33–41	1.6
Ex 5	5.2	3.9–6.9	5.8	13	11–14	1.8	56	50–64	1.8
**SN-38**									
Ex 1	4.9	4.5–5.3	2.3	16	15–17	1.2	52	49–56	1.1
Ex 2	2.1	1.9–2.3	2.7	17	15–20	1.8	69	63–75	1.2
Ex 3	2.3	2.2–2.5	1.5	16	15–17	1.3	58	55–62	1.1
Ex 4	2.1	2.0–2.2	1.5	14	10–19	3.9	57	54–60	0.83
Ex 5	3.0	2.8–3.2	1.8	20	19–21	0.68	63	58–69	1.8

EC50 was operationally defined as the drug concentration at which the fitted dose–response curve reached 50% cell viability relative to the untreated control. Dose–response data from each independent experiment were fitted separately using a four-parameter log-logistic model (LL.4). The 95% confidence interval (CI) of each EC50 estimate was calculated on the logarithmic EC50 scale using the delta method and then back-transformed to the original concentration scale. Residual standard error (RSE) is reported as a measure of residual variation for each fitted curve. Each concentration was measured in four technical replicate wells, and the mean of the four wells was used for curve fitting. Five independent experiments were performed for each cell line.

Figure S2. Western Blot Original image.

(A) GAPDH

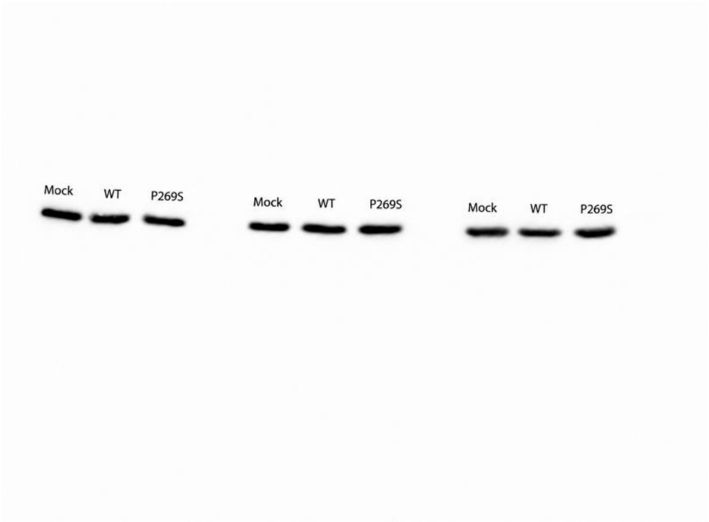

(A) ABCG2

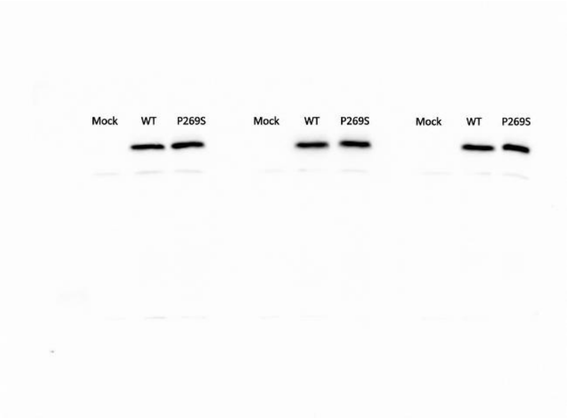

(B) N-Cadherin

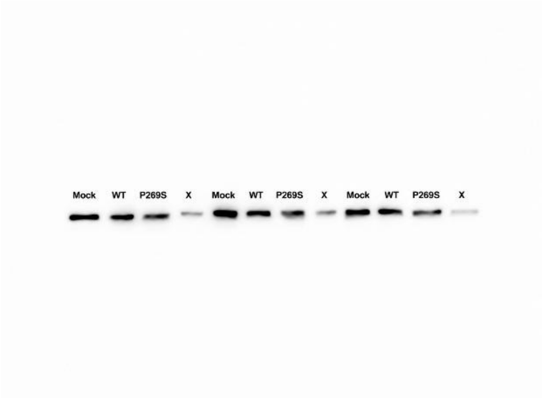

(B) ABCG2

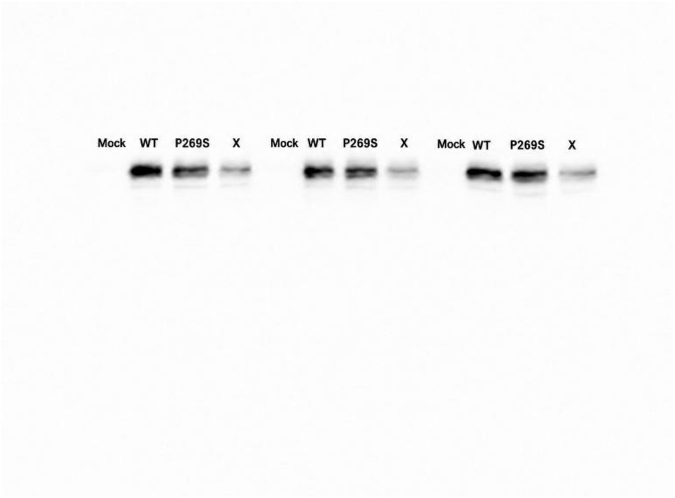

Supplement: Supplementary file 1 [file biomolecules-16-01486-s001.zip › biomolecules-4584694-supplementary.pdf]
